# Supplementary material for: Enhanced kinase translocation reporters for simultaneous real-time measurement of PKA, ERK, and Ca2+
Source: bioRxiv. 2024 Oct 2:2024.09.30.615856. Preprint. [Version 1] doi: 10.1101/2024.09.30.615856 (PMC11475874; doi:10.1101/2024.09.30.615856)
Supplement: Supplement 8 [file NIHPP2024.09.30.615856v1-supplement-8.pdf]

bioRxiv preprint doi: https://doi.org/10.1101/2024.09.30.615856; this version posted October 2, 2024. The copyright holder for this preprint (which was not certified by peer review) is the author/funder, who has granted bioRxiv a license to display the preprint in perpetuity. It is made available under aCC-BY 4.0 International license.

SUPPLEMENTAL INFORMATION

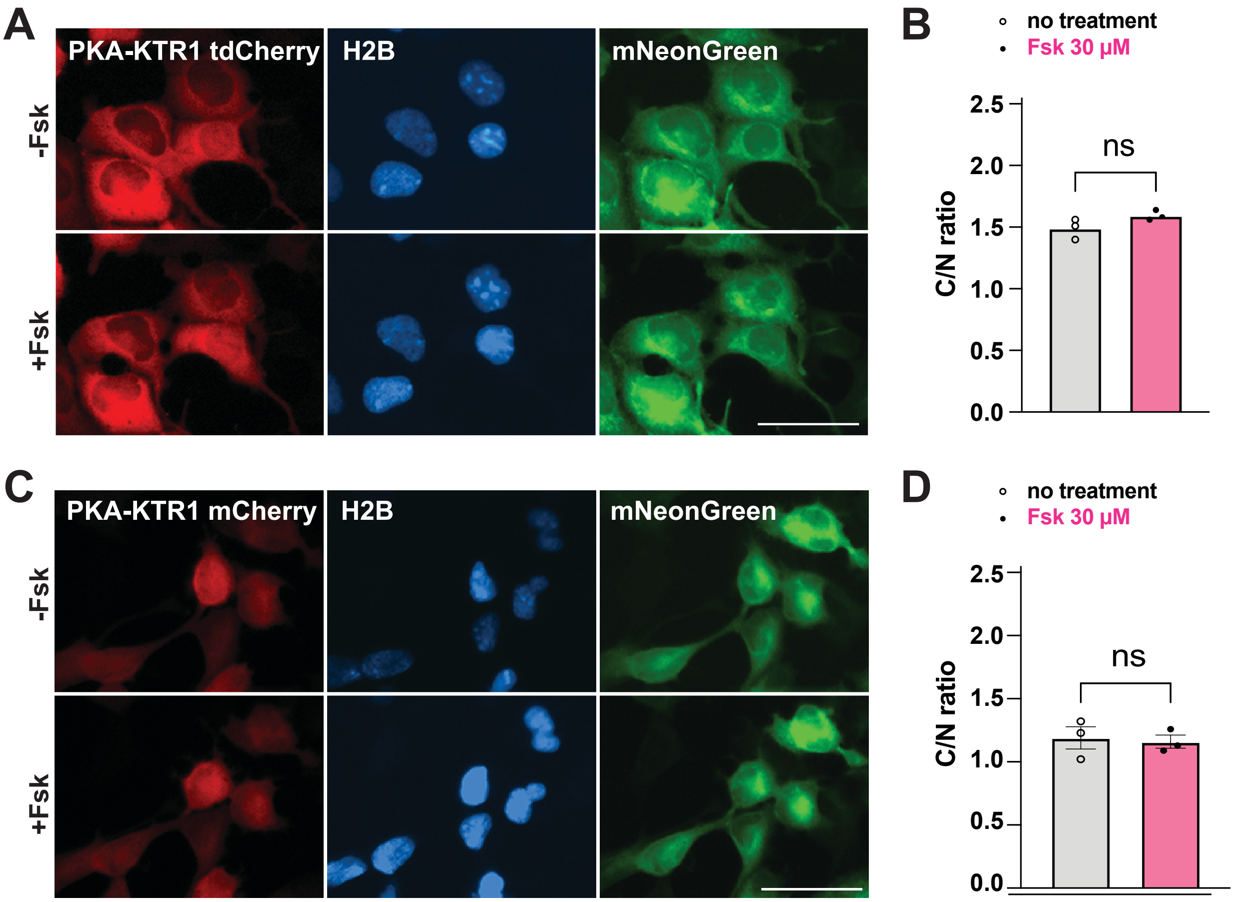

Figure S1. The PKA-KTR1 sensor domain does not drive Fsk-induced protein translocation to the cytoplasm. HEK293 cells were transfected with plasmids designed to co-express H2B-mTagBFP2, mNeonGreen, and (A, B) PKA-KTR1/tdCherry (~64 kDa), or (C, D) PKA-KTR1-mCherry (~32 kDa). (**A**) Fluorescence micrographs of HEK293 expressing cells expressing PKA-KTR1/tdCherry before and after 30 minutes incubation in 30 µM Fsk. Bar, 50 µM. (**B**) Bar plot of PKA-KTR1/tdCherry C/N ratio before and after addition of after 30 minutes incubation in 30 µM Fsk. (**C**) Fluorescence micrographs of HEK293 expressing cells expressing PKA-KTR1/mCherry before and after 30 minutes incubation in 30 µM Fsk. Bar, 50 µM. (**D**) Bar plot of PKA-KTR1/mCherry C/N ratio before and after addition of after 30 minutes incubation in 30 µM Fsk. Data are from a minimum of three independent biological replicates.

bioRxiv preprint doi: https://doi.org/10.1101/2024.09.30.615856; this version posted October 2, 2024. The copyright holder for this preprint (which was not certified by peer review) is the author/funder, who has granted bioRxiv a license to display the preprint in perpetuity. It is made available under aCC-BY 4.0 International license.

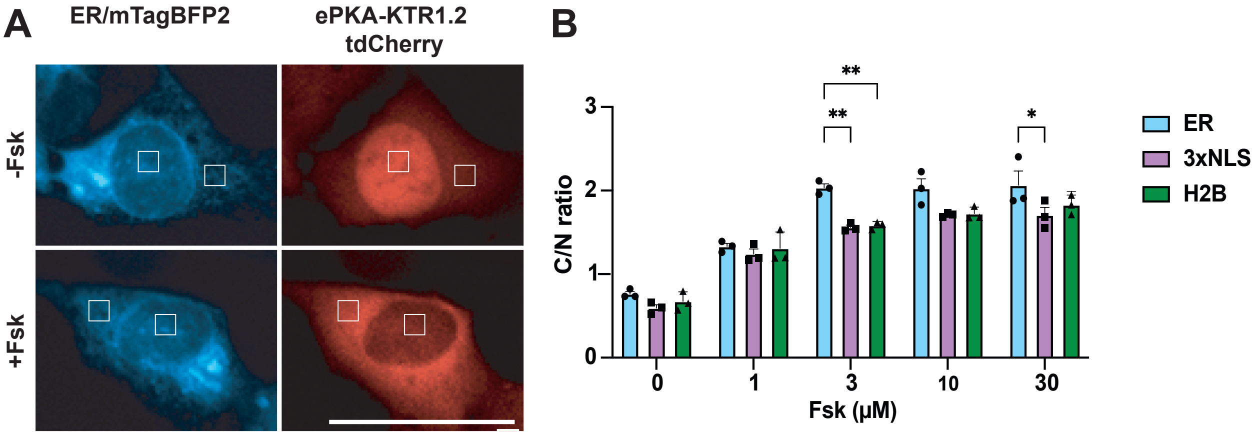

Figure S2. Marking nucleus and cytoplasm with ER-mTagBFP2. (**A**) Fluorescence micrographs of HEK293 cells co-expressing ER-mTagBFP2 and ePKA-KTR1.2/tdCherry. The distribution of ER-mTagBFP2 determines the positioning of 'counting areas' (white boxes) that are then used to count the relative fluorescence intensity of KTR proteins in the nucleus and cytoplasm, with the C/N ratio determined from the average fluorescence brightness of all pixels within each counting area. (**B)** C/N ratios of ePKA-KTR1.2 in HEK293 cells co-expressing (blue bars) the ER-mTagBFP2 marker (ER), (purple bars) the 3xNLS-mTagBFP2 marker (3xNLS), or (green bars) the H2B-mTagBFP2 marker (H2B). Cells were exposed to 0, 1, 3, 10, or 30 uM Fsk for 30 minutes, imaged by fluorescence microscopy, and C/N ratios were calculated from digital images. Data are from a minimum of three independent biological replicates. ANOVA p values are denoted by * <0.05, and ** <0.01.

bioRxiv preprint doi: https://doi.org/10.1101/2024.09.30.615856; this version posted October 2, 2024. The copyright holder for this preprint (which was not certified by peer review) is the author/funder, who has granted bioRxiv a license to display the preprint in perpetuity. It is made available under aCC-BY 4.0 International license.

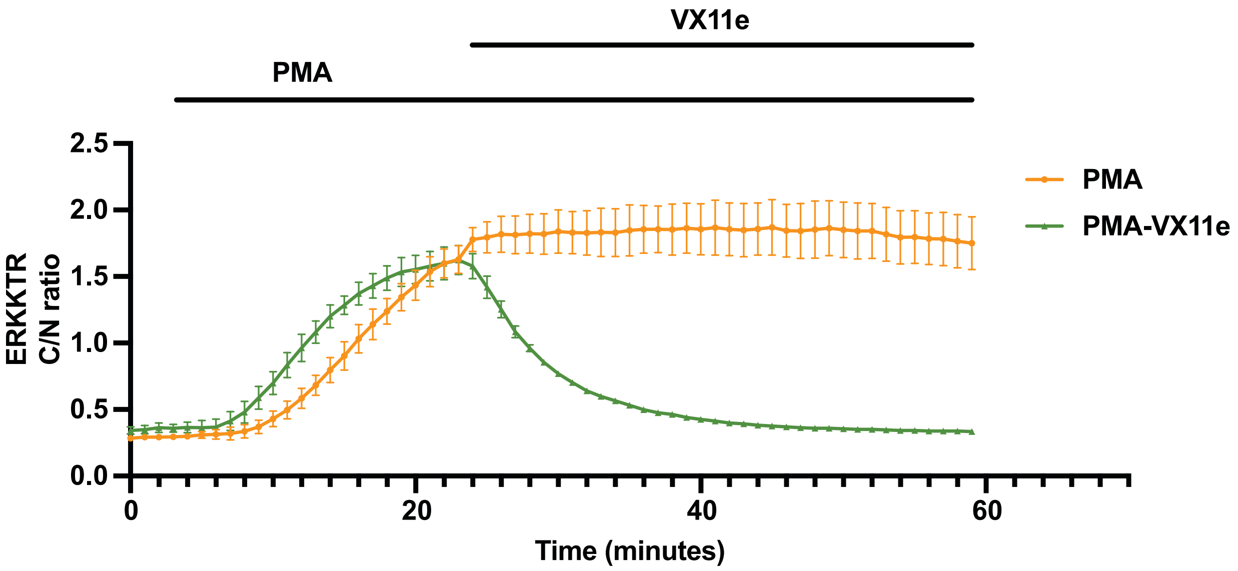

Figure S3. Response of eERK-KTR1.2/emiRFP670 to PMA, and to PMA then VX11e. HEK293 cells expressing eERK-KTR1.2/emiRFP670 were imaged every 60 sec (orange) in response to PMA alone or (green) in response to PMA, followed 20 min later by addition of VX11e. Data are from a minimum of three independent biological replicates.

bioRxiv preprint doi: https://doi.org/10.1101/2024.09.30.615856; this version posted October 2, 2024. The copyright holder for this preprint (which was not certified by peer review) is the author/funder, who has granted bioRxiv a license to display the preprint in perpetuity. It is made available under aCC-BY 4.0 International license.

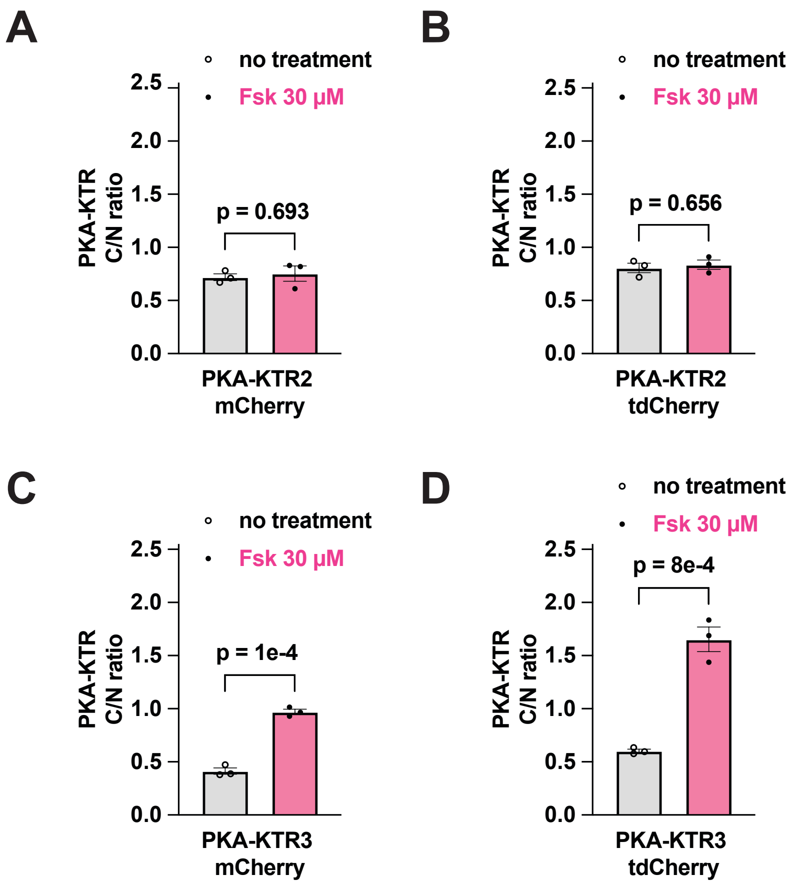

Figure S4. Increasing KTR size triples the dynamic range of PKA-KTR3. HEK293 cells were transfected with plasmid vectors designed to express fusion proteins comprised of the sensor domains of PKA-KTR2(*28*) or PKA-KTR3(*29*) appended to the N-terminus of either mCherry, tdCherry. Two days later, cells were imaged by fluorescence microscopy prior to or after a 30 min. incubation in 30 mM Fsk, followed by calculation of C/N ratios in 10 cells in independent regions of interest (ROIs) from three biological replicates, shown here in bar graph form. (**A, B**) Both PKA-KTR2/mCherry, a ~34 kDa proteins, and PKA-KTR2/tdCherry, a ~64 kDa protein, had resting C/N ratios <1, and neither showed a Fsk-induced translocation to the cytoplasm. (**C**) PKA-KTR3/mCherry, a ~34 kDa protein, displayed a low resting C/N ratio and moved to the cytoplasm in response to Fsk, confirming that the PKA-KTR3 sensor domain is a useful reporter of PKA activity. However, Fsk-induced C/N ratio of PKA-KTR3/mCherry remained <1, and its dynamic range was relatively narrow, only ~2-fold. (**D**) In contrast, we found that PKA-KTR3/tdCherry, ~64 kDa, had a broader dynamic range, ~3-fold, showing once again that increasing KTR size can be sufficient to improve KTR performance characteristics. Data are from a minimum of three independent biological replicates. ANOVA p values are denoted by *** <0.001.

bioRxiv preprint doi: https://doi.org/10.1101/2024.09.30.615856; this version posted October 2, 2024. The copyright holder for this preprint (which was not certified by peer review) is the author/funder, who has granted bioRxiv a license to display the preprint in perpetuity. It is made available under aCC-BY 4.0 International license.

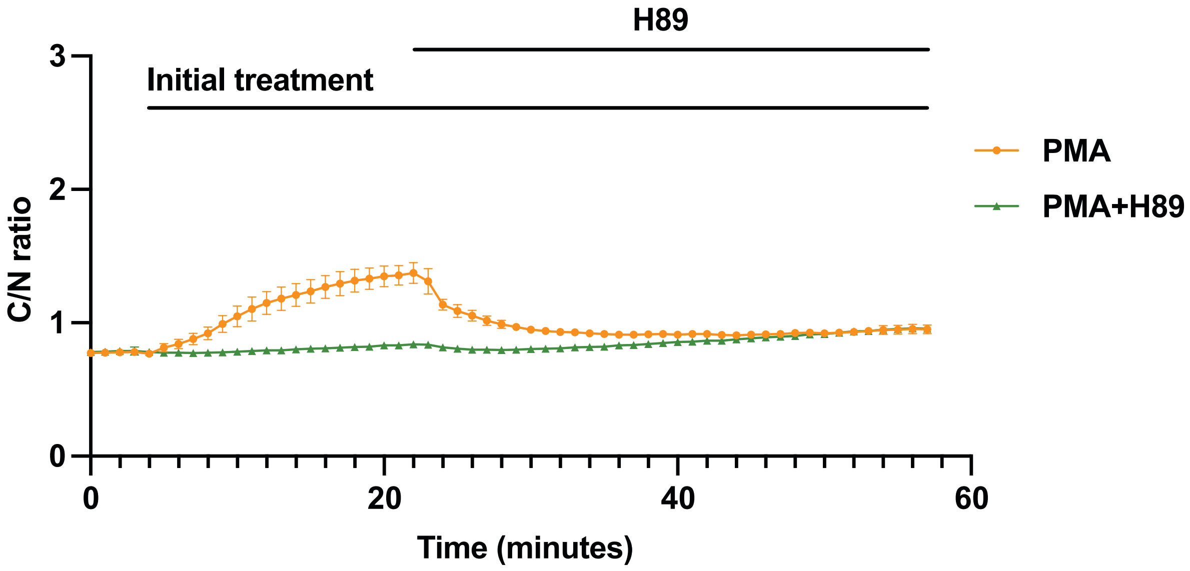

Figure S5. PMA induces a mild activation of PKA. Plot of C/N ratios for ePKA-KTR1.2/tdTomato C/N at every minute in ePKA-KTR1.2/tdTomato-expressing HEK293 cells exposed to (orange) PMA at t = 3 min, followed by the addition of the PKA inhibitor H89 at t = 23, or (green) PMA and H89 at t = 3, followed by addition of H89 again at t = 23 min.

bioRxiv preprint doi: https://doi.org/10.1101/2024.09.30.615856; this version posted October 2, 2024. The copyright holder for this preprint (which was not certified by peer review) is the author/funder, who has granted bioRxiv a license to display the preprint in perpetuity. It is made available under aCC-BY 4.0 International license.

***Table S1. Description, amino acid sequences, vector type, and plasmid number*.** Vectors pC and pLenti were described previously(*62*). Amino acid sequences are represented in single letter code, with bold, black lettering for kinase sensor domains, red lettering for red and infrared fluorescent proteins, blue lettering for mTagBFP2, and black, unbolded lettering for other sequence elements (i.e. linker sequences, histone H2B sequence, C-terminal extensions, signal sequence, ER retrieval signal, and 3xNLS). The notation **|<…>|** denotes the position in the viral 2a peptide where the ribosome fails to make the peptide bond (between the upstream glycine and the downstream proline), resulting in the release of the upstream red or infrared fluorescent protein and the continued translation of the downstream blue fluorescent protein.

<table>
<tr><th>ORF Description</th><th>Deduced Amino Acid Sequences of the ORF</th><th>vector</th><th>plasmid #</th></tr>
<tr><td>PKA-KTR1/tdCherry.2a.H2B-mTagBFP2</td><td>MEMPEEPANSGHSLPPVYIYSPEYVSICDSLVKVPKRASMVNEDEAPSRRKASGQVSSRLERLTLQSSGAPVSKGEEDN
MAIIKEFMRFKVHMEGSVNGHEFEIEGEGEGRPYEGTQTAKLKVTKGGPLPFAWDILSPQFMYGSKAYVKHPADIPDYL
KLSFPEGFKWERVMNFEDGGVVTVTQDSSLQDGEFIYKVKLRGTNFPSDGPVMQKKTMGWEASSERMYPEDGALKGEIK
QRLKLKDGGHYDAEVKTTYKAKKPVQLPGAYNVNIKLDITSHNEDYTIVEQYERAEGRHSTGGMDELYKARGSAGSATT
MVSKGEEDNMAIIKEFMRFKVHMEGSVNGHEFEIEGEGEGRPYEGTQTAKLKVTKGGPLPFAWDILSPQFMYGSKAYVK
HPADIPDYLKLSFPEGFKWERVMNFEDGGVVTVTQDSSLQDGEFIYKVKLRGTNFPSDGPVMQKKTMGWEASSERMYPE
DGALKGEIKQRLKLKDGGHYDAEVKTTYKAKKPVQLPGAYNVNIKLDITSHNEDYTIVEQYERAEGRHSTGGMDELYKA
SGSGATNFSLLKQAGDVEENPG|<....>|PSPAMPEPAKSAPAPKKGSKKAVTKAQKKGGKKRKRSRKESYSIYVYKV
LKQVHPDTGISSKAMGIMNSFVNDIFERIAGEASRLAHYNKRSTITSREIQTAVRLLLPGELAKHAVSEGTKAITKYTS
AKDPGGGSSRVSKGEELIKENMHMKLYMEGTVDNHHFKCTSEGEGKPYEGTQTMRIKVVEGGPLPFAFDILATSFLYGS
KTFINHTQGIPDFFKQSFPEGFTWERVTTYEDGGVLTATQDTSLQDGCLIYNVKIRGVNFTSNGPVMQKKTLGWEAFTE
TLYPADGGLEGRNDMALKLVGGSHLIANAKTTYRSKKPAKNLKMPGVYYVDYRLERIKEANNETYVEQHEVAVARYCDL
PSKLGHKLNGMDELYKGRSPGLNGGSGATNFSLLKQAGDVEENPG</td><td>pC</td><td>S740</td></tr>
<tr><td>PKA-KTR1/mCherry.2a.H2B-mTagBFP2</td><td>MEMPEEPANSGHSLPPVYIYSPEYVSICDSLVKVPKRASMVNEDEAPSRRKASGQVSSRLERLTLQSSGAPVSKGEEDN
MAIIKEFMRFKVHMEGSVNGHEFEIEGEGEGRPYEGTQTAKLKVTKGGPLPFAWDILSPQFMYGSKAYVKHPADIPDYL
KLSFPEGFKWERVMNFEDGGVVTVTQDSSLQDGEFIYKVKLRGTNFPSDGPVMQKKTMGWEASSERMYPEDGALKGEIK
QRLKLKDGGHYDAEVKTTYKAKKPVQLPGAYNVNIKLDITSHNEDYTIVEQYERAEGRHSTGGMDELYKASGSGATNFS
LLKQAGDVEENPG|<....>|PSPAMPEPAKSAPAPKKGSKKAVTKAQKKGGKKRKRSRKESYSIYVYKVLKQVHPDTG
ISSKAMGIMNSFVNDIFERIAGEASRLAHYNKRSTITSREIQTAVRLLLPGELAKHAVSEGTKAITKYTSAKDPGGGSS
RVSKGEELIKENMHMKLYMEGTVDNHHFKCTSEGEGKPYEGTQTMRIKVVEGGPLPFAFDILATSFLYGSKTFINHTQG
IPDFFKQSFPEGFTWERVTTYEDGGVLTATQDTSLQDGCLIYNVKIRGVNFTSNGPVMQKKTLGWEAFTETLYPADGGL
EGRNDMALKLVGGSHLIANAKTTYRSKKPAKNLKMPGVYYVDYRLERIKEANNETYVEQHEVAVARYCDLPSKLGHKLN
GMDELYKGRSPGLNGGSGATNFSLLKQAGDVEENPG</td><td>pC</td><td>S739</td></tr>
<tr><td>ePKA-KTR1.1/tdCherry.2a.H2B-mTagBFP2</td><td>MEMPEEPANSGHSLPPVYIYSPEYVSICDSLVKVPKRASMVNEDEAPSKATRRASLGVSSRLERLTLQSSGAPVSKGEE
DNMAIIKEFMRFKVHMEGSVNGHEFEIEGEGEGRPYEGTQTAKLKVTKGGPLPFAWDILSPQFMYGSKAYVKHPADIPD
YLKLSFPEGFKWERVMNFEDGGVVTVTQDSSLQDGEFIYKVKLRGTNFPSDGPVMQKKTMGWEASSERMYPEDGALKGE
IKQRLKLKDGGHYDAEVKTTYKAKKPVQLPGAYNVNIKLDITSHNEDYTIVEQYERAEGRHSTGGMDELYKARGSAGSA
TTMVSKGEEDNMAIIKEFMRFKVHMEGSVNGHEFEIEGEGEGRPYEGTQTAKLKVTKGGPLPFAWDILSPQFMYGSKAY
VKHPADIPDYLKLSFPEGFKWERVMNFEDGGVVTVTQDSSLQDGEFIYKVKLRGTNFPSDGPVMQKKTMGWEASSERMY
PEDGALKGEIKQRLKLKDGGHYDAEVKTTYKAKKPVQLPGAYNVNIKLDITSHNEDYTIVEQYERAEGRHSTGGMDELY
KASGSGATNFSLLKQAGDVEENPG|<....>|PSPAMPEPAKSAPAPKKGSKKAVTKAQKKGGKKRKRSRKESYSIYVY
KVLKQVHPDTGISSKAMGIMNSFVNDIFERIAGEASRLAHYNKRSTITSREIQTAVRLLLPGELAKHAVSEGTKAITKY
TSAKDPGGGSSRVSKGEELIKENMHMKLYMEGTVDNHHFKCTSEGEGKPYEGTQTMRIKVVEGGPLPFAFDILATSFLY
GSKTFINHTQGIPDFFKQSFPEGFTWERVTTYEDGGVLTATQDTSLQDGCLIYNVKIRGVNFTSNGPVMQKKTLGWEAF
TETLYPADGGLEGRNDMALKLVGGSHLIANAKTTYRSKKPAKNLKMPGVYYVDYRLERIKEANNETYVEQHEVAVARYC
DLPSKLGHKLNGMDELYKGRSPGLNGGSGATNFSLLKQAGDVEENPG</td><td>pC</td><td>S741</td></tr>
<tr><td>ePKA-KTR1.2/tdCherry.2a.H2B-mTagBFP2</td><td>MEMPEEPANSGHSLPPVYIYSPEYVSICDSLVKVPKRASMVNEDEAPSKRRASLGVSSRLERLTLQSSGAPVSKGEEDN
MAIIKEFMRFKVHMEGSVNGHEFEIEGEGEGRPYEGTQTAKLKVTKGGPLPFAWDILSPQFMYGSKAYVKHPADIPDYL
KLSFPEGFKWERVMNFEDGGVVTVTQDSSLQDGEFIYKVKLRGTNFPSDGPVMQKKTMGWEASSERMYPEDGALKGEIK
QRLKLKDGGHYDAEVKTTYKAKKPVQLPGAYNVNIKLDITSHNEDYTIVEQYERAEGRHSTGGMDELYKARGSAGSATT
MVSKGEEDNMAIIKEFMRFKVHMEGSVNGHEFEIEGEGEGRPYEGTQTAKLKVTKGGPLPFAWDILSPQFMYGSKAYVK
HPADIPDYLKLSFPEGFKWERVMNFEDGGVVTVTQDSSLQDGEFIYKVKLRGTNFPSDGPVMQKKTMGWEASSERMYPE
DGALKGEIKQRLKLKDGGHYDAEVKTTYKAKKPVQLPGAYNVNIKLDITSHNEDYTIVEQYERAEGRHSTGGMDELYKA
SGSGATNFSLLKQAGDVEENPG|<....>|PSPAMPEPAKSAPAPKKGSKKAVTKAQKKGGKKRKRSRKESYSIYVYKV
LKQVHPDTGISSKAMGIMNSFVNDIFERIAGEASRLAHYNKRSTITSREIQTAVRLLLPGELAKHAVSEGTKAITKYTS
AKDPGGGSSRVSKGEELIKENMHMKLYMEGTVDNHHFKCTSEGEGKPYEGTQTMRIKVVEGGPLPFAFDILATSFLYGS
KTFINHTQGIPDFFKQSFPEGFTWERVTTYEDGGVLTATQDTSLQDGCLIYNVKIRGVNFTSNGPVMQKKTLGWEAFTE</td><td>pC</td><td>S467</td></tr>
</table>

bioRxiv preprint doi: https://doi.org/10.1101/2024.09.30.615856; this version posted October 2, 2024. The copyright holder for this preprint (which was not certified by peer review) is the author/funder, who has granted bioRxiv a license to display the preprint in perpetuity. It is made available under aCC-BY 4.0 International license.

<table>
<tr><td></td><td>TLYPADGGLEGRNDMALKLVGGSHLIANAKTTYRSKKPAKNLKMPGVYYVDYRLERIKEANNETYVEQHEVAVARYCDLPSKLGHKLNGMDELYKGRSPGLNGGSGATNFSLLKQAGDVEENPG</td><td></td><td></td></tr>
<tr><td>ePKA-KTR1.3/tdCherry.2a.H2B-mTagBFP2</td><td>MEMPEEPANSGHSLPPVYIYSPEYVSICDSLVKVPKRASMVNEDEAPAKRRASLGVSSRLERLTLQSSGAPVSKGEEDNMAIIKEFMRFKVHMEGSVNGHEFEIEGEGEGRPYEGTQTAKLKVTKGGPLPFAWDILSPQFMYGSKAYVKHPADIPDYLKLSFPEGFKWERVMNFEDGGVVTVTQDSSLQDGEFIYKVKLRGTNFPSDGPVMQKKTMGWEASSERMYPEDGALKGEIKQRLKLKDGGHYDAEVKTTYKAKKPVQLPGAYNVNIKLDITSHNEDYTIVEQYERAEGRHSTGGMDELYKARGSAGSATTMVSKGEEDNMAIIKEFMRFKVHMEGSVNGHEFEIEGEGEGRPYEGTQTAKLKVTKGGPLPFAWDILSPQFMYGSKAYVKHPADIPDYLKLSFPEGFKWERVMNFEDGGVVTVTQDSSLQDGEFIYKVKLRGTNFPSDGPVMQKKTMGWEASSERMYPEDGALKGEIKQRLKLKDGGHYDAEVKTTYKAKKPVQLPGAYNVNIKLDITSHNEDYTIVEQYERAEGRHSTGGMDELYKASGSGATNFSLLKQAGDVEENPG|<....>|PSPAMPEPAKSAPAPKKGSKKAVTKAQKKGGKKRKRSRKESYSIYVYKVLKQVHPDTGISSKAMGIMNSFVNDIFERIAGEASRLAHYNKRSTITSREIQTAVRLLLPGELAKHAVSEGTKAITKYTSAKDPGGGSSRVSKGEELIKENMHMKLYMEGTVDNHHFKCTSEGEGKPYEGTQTMRIKVVEGGPLPFAFDILATSFLYGSKTFINHTQGIPDFFKQSFPEGFTWERVTTYEDGGVLTATQDTSLQDGCLIYNVKIRGVNFTSNGPVMQKKTLGWEAFTETLYPADGGLEGRNDMALKLVGGSHLIANAKTTYRSKKPAKNLKMPGVYYVDYRLERIKEANNETYVEQHEVAVARYCDLPSKLGHKLNGMDELYKGRSPGLNGGSGATNFSLLKQAGDVEENPG</td><td>pC</td><td>S514</td></tr>
<tr><td>ePKA-KTR1.4/tdCherry.2a.H2B-mTagBFP2</td><td>MEMPEEPANSGHSLPPVYIYSPEYVSICDSLVKVPKRASMVNEEDEAPAKRRASLGVSSRLERLTLQSSGAPVSKGEEDNMAIIKEFMRFKVHMEGSVNGHEFEIEGEGEGRPYEGTQTAKLKVTKGGPLPFAWDILSPQFMYGSKAYVKHPADIPDYLKLSFPEGFKWERVMNFEDGGVVTVTQDSSLQDGEFIYKVKLRGTNFPSDGPVMQKKTMGWEASSERMYPEDGALKGEIKQRLKLKDGGHYDAEVKTTYKAKKPVQLPGAYNVNIKLDITSHNEDYTIVEQYERAEGRHSTGGMDELYKARGSAGSATTMVSKGEEDNMAIIKEFMRFKVHMEGSVNGHEFEIEGEGEGRPYEGTQTAKLKVTKGGPLPFAWDILSPQFMYGSKAYVKHPADIPDYLKLSFPEGFKWERVMNFEDGGVVTVTQDSSLQDGEFIYKVKLRGTNFPSDGPVMQKKTMGWEASSERMYPEDGALKGEIKQRLKLKDGGHYDAEVKTTYKAKKPVQLPGAYNVNIKLDITSHNEDYTIVEQYERAEGRHSTGGMDELYKASGSGATNFSLLKQAGDVEENPG|<....>|PSPAMPEPAKSAPAPKKGSKKAVTKAQKKGGKKRKRSRKESYSIYVYKVLKQVHPDTGISSKAMGIMNSFVNDIFERIAGEASRLAHYNKRSTITSREIQTAVRLLLPGELAKHAVSEGTKAITKYTSAKDPGGGSSRVSKGEELIKENMHMKLYMEGTVDNHHFKCTSEGEGKPYEGTQTMRIKVVEGGPLPFAFDILATSFLYGSKTFINHTQGIPDFFKQSFPEGFTWERVTTYEDGGVLTATQDTSLQDGCLIYNVKIRGVNFTSNGPVMQKKTLGWEAFTETLYPADGGLEGRNDMALKLVGGSHLIANAKTTYRSKKPAKNLKMPGVYYVDYRLERIKEANNETYVEQHEVAVARYCDLPSKLGHKLNGMDELYKGRSPGLNGGSGATNFSLLKQAGDVEENPG</td><td>pC</td><td>S515</td></tr>
<tr><td>ePKA-KTR1.5/tdCherry.2a.H2B-mTagBFP2</td><td>MEMPEEPANSGHSLPPVYIYSPEYVSICDSLVKVPKRASMVNEDEAPSKKRRASLGVSSRLERLTLQSSGAPVSKGEEDNMAIIKEFMRFKVHMEGSVNGHEFEIEGEGEGRPYEGTQTAKLKVTKGGPLPFAWDILSPQFMYGSKAYVKHPADIPDYLKLSFPEGFKWERVMNFEDGGVVTVTQDSSLQDGEFIYKVKLRGTNFPSDGPVMQKKTMGWEASSERMYPEDGALKGEIKQRLKLKDGGHYDAEVKTTYKAKKPVQLPGAYNVNIKLDITSHNEDYTIVEQYERAEGRHSTGGMDELYKARGSAGSATTMVSKGEEDNMAIIKEFMRFKVHMEGSVNGHEFEIEGEGEGRPYEGTQTAKLKVTKGGPLPFAWDILSPQFMYGSKAYVKHPADIPDYLKLSFPEGFKWERVMNFEDGGVVTVTQDSSLQDGEFIYKVKLRGTNFPSDGPVMQKKTMGWEASSERMYPEDGALKGEIKQRLKLKDGGHYDAEVKTTYKAKKPVQLPGAYNVNIKLDITSHNEDYTIVEQYERAEGRHSTGGMDELYKASGSGATNFSLLKQAGDVEENPG|<....>|PSPAMPEPAKSAPAPKKGSKKAVTKAQKKGGKKRKRSRKESYSIYVYKVLKQVHPDTGISSKAMGIMNSFVNDIFERIAGEASRLAHYNKRSTITSREIQTAVRLLLPGELAKHAVSEGTKAITKYTSAKDPGGGSSRVSKGEELIKENMHMKLYMEGTVDNHHFKCTSEGEGKPYEGTQTMRIKVVEGGPLPFAFDILATSFLYGSKTFINHTQGIPDFFKQSFPEGFTWERVTTYEDGGVLTATQDTSLQDGCLIYNVKIRGVNFTSNGPVMQKKTLGWEAFTETLYPADGGLEGRNDMALKLVGGSHLIANAKTTYRSKKPAKNLKMPGVYYVDYRLERIKEANNETYVEQHEVAVARYCDLPSKLGHKLNGMDELYKGRSPGLNGGSGATNFSLLKQAGDVEENPG</td><td>pC</td><td>S742</td></tr>
<tr><td>ePKA-KTR1.2/mCherry.2a.ER-mTagBFP2</td><td>MEMPEEPANSGHSLPPVYIYSPEYVSICDSLVKVPKRASMVNEDEAPSKRRASLGVSSRLERLTLQSSGAPVSKGEEDNMAIIKEFMRFKVHMEGSVNGHEFEIEGEGEGRPYEGTQTAKLKVTKGGPLPFAWDILSPQFMYGSKAYVKHPADIPDYLKLSFPEGFKWERVMNFEDGGVVTVTQDSSLQDGEFIYKVKLRGTNFPSDGPVMQKKTMGWEASSERMYPEDGALKGEIKQRLKLKDGGHYDAEVKTTYKAKKPVQLPGAYNVNIKLDITSHNEDYTIVEQYERAEGRHSTGGMDELYKASGSGATNFSLLKQAGDVEENPG|<....>|PMKLSLVAAMLLLLSAARAVSKGEELIKENMHMKLYMEGTVDNHHFKCTSEGEGKPYEGTQTMRIKVVEGGPLPFAFDILATSFLYGSKTFINHTQGIPDFFKQSFPEGFTWERVTTYEDGGVLTATQDTSLQDGCLIYNVKIRGVNFTSNGPVMQKKTLGWEAFTETLYPADGGLEGRNDMALKLVGGSHLIANAKTTYRSKKPAKNLKMPGVYYVDYRLERIKEANNETYVEQHEVAVARYCDLPSKLGHKLNKDEL</td><td>pLenti</td><td>YG61</td></tr>
<tr><td>ePKA-KTR1.4/mCherry.2a.ER-mTagBFP2</td><td>MEMPEEPANSGHSLPPVYIYSPEYVSICDSLVKVPKRASMVNEEDEAPAKRRASLGVSSRLERLTLQSSGAPVSKGEEDNMAIIKEFMRFKVHMEGSVNGHEFEIEGEGEGRPYEGTQTAKLKVTKGGPLPFAWDILSPQFMYGSKAYVKHPADIPDYLKLSFPEGFKWERVMNFEDGGVVTVTQDSSLQDGEFIYKVKLRGTNFPSDGPVMQKKTMGWEASSERMYPEDGALKGEIKQRLKLKDGGHYDAEVKTTYKAKKPVQLPGAYNVNIKLDITSHNEDYTIVEQYERAEGRHSTGGMDELYKASGSGATNFSLLKQAGDVEENPG|<....>|PMKLSLVAAMLLLLSAARAVSKGEELIKENMHMKLYMEGTVDNHHFKCTSEGEGKPYEGTQTMRIKVVEGGPLPFAFDILATSFLYGSKTFINHTQGIPDFFKQSFPEGFTWERVTTYEDGGVLTATQDTSLQDGCLIYNVKIRGVNFTSNGPVMQKKTLGWEAFTETLYPADGGLEGRNDMALKLVGGSHLIANAKTTYRSKKPAKNLKMPGVYYVDYRLERIKEANNETYVEQHEVAVARYCDLPSKLGHKLNKDEL</td><td>pLenti</td><td>YG65</td></tr>
<tr><td>ePKA-KTR1.4/tdCherry.2a.ER-mTagBFP2</td><td>MEMPEEPANSGHSLPPVYIYSPEYVSICDSLVKVPKRASMVNEEDEAPAKRRASLGVSSRLERLTLQSSGAPVSKGEEDNMAIIKEFMRFKVHMEGSVNGHEFEIEGEGEGRPYEGTQTAKLKVTKGGPLPFAWDILSPQFMYGSKAYVKHPADIPDYLKLSFPEGFKWERVMNFEDGGVVTVTQDSSLQDGEFIYKVKLRGTNFPSDGPVMQKKTMGWEASSERMYPEDGALKGEIKQRLKLKDGGHYDAEVKTTYKAKKPVQLPGAYNVNIKLDITSHNEDYTIVEQYERAEGRHSTGGMDELYKARGSAGSATTMVSKGEEDNMAIIKEFMRFKVHMEGSVNGHEFEIEGEGEGRPYEGTQTAKLKVTKGGPLPFAWDILSPQFMYGSKAYVKHPADIPDYLKLSFPEGFKWERVMNFEDGGVVTVTQDSSLQDGEFIYKVKLRGTNFPSDGPVMQKKTMGWEASSERMYPEDGALKGEIKQRLKLKDGGHYDAEVKTTYKAKKPVQLPGAYNVNIKLDITSHNEDYTIVEQYERAEGRHSTGGMDELYKASGSGATNFSLLKQAGDVEENPG|<....>|PMKLSLVAAMLLLLSAARAVSKGEELIKENMHMKLYMEGTVDNHHFKCTSEGEGKPYEGTQTMRIKVVEGGPLPFAFDILATSFLYGSKTFINHTQGIPDFFKQSFPEGFTWERVTTYEDGGVLTATQDTSLQDGCLIYNVKIRGVNFTSNGPVMQKKTLGWEAFTETLYPADGGLEGRNDMALKLVGGSHLIANAKTTYRSKKPAKNLKMPGVYYVDYRLERIKEANNETYVEQHEVAVARYCDLPSKLGHKLNKDEL</td><td>pLenti</td><td>YG67</td></tr>
<tr><td>ePKA-KTR1.2/mScar</td><td>MEMPEEPANSGHSLPPVYIYSPEYVSICDSLVKVPKRASMVNEDEAPSKRRASLGVSSRLERLTLQSSGAPVSKGEAVIKEFMRFKVHMEGSMNGHEFEIEGEGEGRPYEGTQTAKLKVTKGGPLPFSWDILSPQFMYGSRAFIKHPADIPDYYKQSFPEGFKWERVMNFEDGGAVTVTQDTSLEDGTLIYKVKLRGTNFPPDGPVMQKKTMGWEASTERLYPEDGVLKGDIKMALR</td><td>pLe</td><td>YG8</td></tr>
</table>

bioRxiv preprint doi: https://doi.org/10.1101/2024.09.30.615856; this version posted October 2, 2024. The copyright holder for this preprint (which was not certified by peer review) is the author/funder, who has granted bioRxiv a license to display the preprint in perpetuity. It is made available under aCC-BY 4.0 International license.

<table>
<tr><td>let-I.2a.ER-mTagBFP2</td><td>LKDGGRYLADFKTTYKAKKPVQMPGAYNVDRKLDITSHNEDYTVVEQYERSEGRHSTGGMDELYKASGSGATNFSLLKQAGDVEENPG|<....>|PMKLSLVAAMLLLLSAARAVSKGEELIKENMHMKLYMEGTVDNHHFKCTSEGEGKPYEGTQTMRIKVVEGGPLPFAFDILATSFLYGSKTFINHTQGIPDFFKQSFPEGFTWERVTTYEDGGVLTATQDTSLQDGCLIYNVKIRGVNFTSNGPVMQKKTLGWEAFTETLYPADGGLEGRNDMALKLVGGSHLIANAKTTYRSKKPAKNLKMPGVYYVDYRLERIKEANNETYVEQHEVAVARYCDLPSKLGHKLNKDEL</td><td>nti</td><td></td></tr>
<tr><td>ePKA-KTR1.2/mScarlet-I/BCR.2a.ER-mTagBFP2</td><td>MEMPEEPANSGHSLPPVYIYSPEYVSICDSLVKVPKRASMVNEDEAPSKRRASLGVSSRLERLTLQSSGAPVSKGEAVIKEFMRFKVHMEGSMNGHEFEIEGEGEGRPYEGTQTAKLKVTKGGPLPFSWDILSPQFMYGSRAFIKHPADIPDYYKQSFPEGFKWERVMNFEDGGAVTVTQDTSLEDGTLIYKVKLRGTNFPPDGPVMQKKTMGWEASTERLYPEDGVLKGDIKMALRLKDGGRYLADFKTTYKAKKPVQMPGAYNVDRKLDITSHNEDYTVVEQYERSEGRHSTGGMDELYKGSGSAGGSAGGSDIEQELERAKASIRRLEQEVNQERSRMAYLQTLLAKGGSAGGSASGSGATNFSLLKQAGDVEENPG|<....>|PMKLSLVAAMLLLLSAARAVSKGEELIKENMHMKLYMEGTVDNHHFKCTSEGEGKPYEGTQTMRIKVVEGGPLPFAFDILATSFLYGSKTFINHTQGIPDFFKQSFPEGFTWERVTTYEDGGVLTATQDTSLQDGCLIYNVKIRGVNFTSNGPVMQKKTLGWEAFTETLYPADGGLEGRNDMALKLVGGSHLIANAKTTYRSKKPAKNLKMPGVYYVDYRLERIKEANNETYVEQHEVAVARYCDLPSKLGHKLNKDEL</td><td>pLenti</td><td>YG18</td></tr>
<tr><td>ERK-KTR1/tdCherry.2a.H2B-mTagBFP2</td><td>MKGRKPRDLELPLSPSLLGGQGPERTPGSGTSSGLQAPGPALSPSKRSGLEDPATPSKKPRTPSVSSRLERLTLQSSFQFPSGAPVSKGEEDNMAIIKEFMRFKVHMEGSVNGHEFEIEGEGEGRPYEGTQTAKLKVTKGGPLPFAWDILSPQFMYGSKAYVKHPADIPDYLKLSFPEGFKWERVMNFEDGGVVTVTQDSSLQDGEFIYKVKLRGTNFPSDGPVMQKKTMGWEASSERMYPEDGALKGEIKQRLKLKDGGHYDAEVKTTYKAKKPVQLPGAYNVNIKLDITSHNEDYTIVEQYERAEGRHSTGGMDELYKARGSAGSATTMVSKGEEDNMAIIKEFMRFKVHMEGSVNGHEFEIEGEGEGRPYEGTQTAKLKVTKGGPLPFAWDILSPQFMYGSKAYVKHPADIPDYLKLSFPEGFKWERVMNFEDGGVVTVTQDSSLQDGEFIYKVKLRGTNFPSDGPVMQKKTMGWEASSERMYPEDGALKGEIKQRLKLKDGGHYDAEVKTTYKAKKPVQLPGAYNVNIKLDITSHNEDYTIVEQYERAEGRHSTGGMDELYKASGSGATNFSLLKQAGDVEENPG|<....>|PSPAMPEPAKSAPAPKKGSKKAVTKAQKKGGKKRKRSRKESYSIYVYKVLKQVHPDTGISSKAMGIMNSFVNDIFERIAGEASRLAHYNKRSTITSREIQTAVRLLLPGELAKHAVSEGTKAITKYTSAKDPGGGSSRVSKGEELIKENMHMKLYMEGTVDNHHFKCTSEGEGKPYEGTQTMRIKVVEGGPLPFAFDILATSFLYGSKTFINHTQGIPDFFKQSFPEGFTWERVTTYEDGGVLTATQDTSLQDGCLIYNVKIRGVNFTSNGPVMQKKTLGWEAFTETLYPADGGLEGRNDMALKLVGGSHLIANAKTTYRSKKPAKNLKMPGVYYVDYRLERIKEANNETYVEQHEVAVARYCDLPSKLGHKLNGMDELYKGRSPGLNGGSGATNFSLLKQAGDVEENPG</td><td>pC</td><td>S468</td></tr>
<tr><td>eERK-KTR1.1/tdCherry.2a.H2B-mTagBFP2</td><td>MKGRKPRDLELPLSPSLLGGQGPERTPGSGTSSGLQAPGPALSPSKRSGLEDEPATPSKKPRTPSVSSRLERLTLQSSFQFPSGAPVSKGEEDNMAIIKEFMRFKVHMEGSVNGHEFEIEGEGEGRPYEGTQTAKLKVTKGGPLPFAWDILSPQFMYGSKAYVKHPADIPDYLKLSFPEGFKWERVMNFEDGGVVTVTQDSSLQDGEFIYKVKLRGTNFPSDGPVMQKKTMGWEASSERMYPEDGALKGEIKQRLKLKDGGHYDAEVKTTYKAKKPVQLPGAYNVNIKLDITSHNEDYTIVEQYERAEGRHSTGGMDELYKARGSAGSATTMVSKGEEDNMAIIKEFMRFKVHMEGSVNGHEFEIEGEGEGRPYEGTQTAKLKVTKGGPLPFAWDILSPQFMYGSKAYVKHPADIPDYLKLSFPEGFKWERVMNFEDGGVVTVTQDSSLQDGEFIYKVKLRGTNFPSDGPVMQKKTMGWEASSERMYPEDGALKGEIKQRLKLKDGGHYDAEVKTTYKAKKPVQLPGAYNVNIKLDITSHNEDYTIVEQYERAEGRHSTGGMDELYKASGSGATNFSLLKQAGDVEENPG|<....>|PSPAMPEPAKSAPAPKKGSKKAVTKAQKKGGKKRKRSRKESYSIYVYKVLKQVHPDTGISSKAMGIMNSFVNDIFERIAGEASRLAHYNKRSTITSREIQTAVRLLLPGELAKHAVSEGTKAITKYTSAKDPGGGSSRVSKGEELIKENMHMKLYMEGTVDNHHFKCTSEGEGKPYEGTQTMRIKVVEGGPLPFAFDILATSFLYGSKTFINHTQGIPDFFKQSFPEGFTWERVTTYEDGGVLTATQDTSLQDGCLIYNVKIRGVNFTSNGPVMQKKTLGWEAFTETLYPADGGLEGRNDMALKLVGGSHLIANAKTTYRSKKPAKNLKMPGVYYVDYRLERIKEANNETYVEQHEVAVARYCDLPSKLGHKLNGMDELYKGRSPGLNGGSGATNFSLLKQAGDVEENPG</td><td>pC</td><td>S712</td></tr>
<tr><td>eERK-KTR1.2/tdCherry.2a.H2B-mTagBFP2</td><td>MKGRKPRDLELPLSPSLLGGQGPERTPGSGTSSGLQAPGPALSPSKRSGLEDDEPATPSKKPRTPSVSSRLERLTLQSSFQFPSGAPVSKGEEDNMAIIKEFMRFKVHMEGSVNGHEFEIEGEGEGRPYEGTQTAKLKVTKGGPLPFAWDILSPQFMYGSKAYVKHPADIPDYLKLSFPEGFKWERVMNFEDGGVVTVTQDSSLQDGEFIYKVKLRGTNFPSDGPVMQKKTMGWEASSERMYPEDGALKGEIKQRLKLKDGGHYDAEVKTTYKAKKPVQLPGAYNVNIKLDITSHNEDYTIVEQYERAEGRHSTGGMDELYKARGSAGSATTMVSKGEEDNMAIIKEFMRFKVHMEGSVNGHEFEIEGEGEGRPYEGTQTAKLKVTKGGPLPFAWDILSPQFMYGSKAYVKHPADIPDYLKLSFPEGFKWERVMNFEDGGVVTVTQDSSLQDGEFIYKVKLRGTNFPSDGPVMQKKTMGWEASSERMYPEDGALKGEIKQRLKLKDGGHYDAEVKTTYKAKKPVQLPGAYNVNIKLDITSHNEDYTIVEQYERAEGRHSTGGMDELYKASGSGATNFSLLKQAGDVEENPG|<....>|PSPAMPEPAKSAPAPKKGSKKAVTKAQKKGGKKRKRSRKESYSIYVYKVLKQVHPDTGISSKAMGIMNSFVNDIFERIAGEASRLAHYNKRSTITSREIQTAVRLLLPGELAKHAVSEGTKAITKYTSAKDPGGGSSRVSKGEELIKENMHMKLYMEGTVDNHHFKCTSEGEGKPYEGTQTMRIKVVEGGPLPFAFDILATSFLYGSKTFINHTQGIPDFFKQSFPEGFTWERVTTYEDGGVLTATQDTSLQDGCLIYNVKIRGVNFTSNGPVMQKKTLGWEAFTETLYPADGGLEGRNDMALKLVGGSHLIANAKTTYRSKKPAKNLKMPGVYYVDYRLERIKEANNETYVEQHEVAVARYCDLPSKLGHKLNGMDELYKGRSPGLNGGSGATNFSLLKQAGDVEENPG</td><td>pC</td><td>S713</td></tr>
<tr><td>eERK-KTR1.3/tdCherry.2a.H2B-mTagBFP2</td><td>MKGRKPRDLELPLSPSLLGGQGPERTPGSGTSSGLQAPGPALSPSKRPGLEDDEPATPSKKPRTPSVSSRLERLTLQSSFQFPSGAPVSKGEEDNMAIIKEFMRFKVHMEGSVNGHEFEIEGEGEGRPYEGTQTAKLKVTKGGPLPFAWDILSPQFMYGSKAYVKHPADIPDYLKLSFPEGFKWERVMNFEDGGVVTVTQDSSLQDGEFIYKVKLRGTNFPSDGPVMQKKTMGWEASSERMYPEDGALKGEIKQRLKLKDGGHYDAEVKTTYKAKKPVQLPGAYNVNIKLDITSHNEDYTIVEQYERAEGRHSTGGMDELYKARGSAGSATTMVSKGEEDNMAIIKEFMRFKVHMEGSVNGHEFEIEGEGEGRPYEGTQTAKLKVTKGGPLPFAWDILSPQFMYGSKAYVKHPADIPDYLKLSFPEGFKWERVMNFEDGGVVTVTQDSSLQDGEFIYKVKLRGTNFPSDGPVMQKKTMGWEASSERMYPEDGALKGEIKQRLKLKDGGHYDAEVKTTYKAKKPVQLPGAYNVNIKLDITSHNEDYTIVEQYERAEGRHSTGGMDELYKASGSGATNFSLLKQAGDVEENPG|<....>|PSPAMPEPAKSAPAPKKGSKKAVTKAQKKGGKKRKRSRKESYSIYVYKVLKQVHPDTGISSKAMGIMNSFVNDIFERIAGEASRLAHYNKRSTITSREIQTAVRLLLPGELAKHAVSEGTKAITKYTSAKDPGGGSSRVSKGEELIKENMHMKLYMEGTVDNHHFKCTSEGEGKPYEGTQTMRIKVVEGGPLPFAFDILATSFLYGSKTFINHTQGIPDFFKQSFPEGFTWERVTTYEDGGVLTATQDTSLQDGCLIYNVKIRGVNFTSNGPVMQKKTLGWEAFTETLYPADGGLEGRNDMALKLVGGSHLIANAKTTYRSKKPAKNLKMPGVYYVDYRLERIKEANNETYVEQHEVAVARYCDLPSKLGHKLNGMDELYKGRSPGLNGGSGATNFSLLKQAGDVEENPG</td><td>pC</td><td>S765</td></tr>
<tr><td>eERK-KTR1.2/mScarlet-I.2a.E</td><td>MKGRKPRDLELPLSPSLLGGQGPERTPGSGTSSGLQAPGPALSPSKRSGLEDDEPATPSKKPRTPSVSSRLERLTLQSSFQFPSGAPVSKGEAVIKEFMRFKVHMEGSMNGHEFEIEGEGEGRPYEGTQTAKLKVTKGGPLPFSWDILSPQFMYGSRAFIKHPADIPDYYKQSFPEGFKWERVMNFEDGGAVTVTQDTSLEDGTLIYKVKLRGTNFPPDGPVMQKKTMGWEASTERLYPEDGVLKGDIKMALRLKDGGRYLADFKTTYKAKKPVQMPGAYNVDRKLDITSHNEDYTVVEQYERSEGRHSTGGMDELYKASGSGATNFSLLKQAGDVEENPG|<....>|PMKLSLVAAMLLLLSAARAVSKGEELIKENMHMKLYMEGTVDNHHF</td><td>pLen</td><td>S940</td></tr>
</table>

bioRxiv preprint doi: https://doi.org/10.1101/2024.09.30.615856; this version posted October 2, 2024. The copyright holder for this preprint (which was not certified by peer review) is the author/funder, who has granted bioRxiv a license to display the preprint in perpetuity. It is made available under aCC-BY 4.0 International license.

<table>
<tr><td>R-mTagBFP2</td><td>KCTSEGEGKPYEGTQTMRIKVVEGGPLPFAFDILATSFLYGSKTFINHTQGIPDFFKQSFPEGFTWERVTTYEDGGVLTATQDTSLQDGCLIYNVKIRGVNFTSNGPVMQKKTLGWEAFTETLYPADGGLEGRNDMALKLVGGSHLIANAKTTYRSKKPAKNLKMPGVYYVDYRLERIKEANNETYVEQHEVAVARYCDLPSKLGHKLNKDEL</td><td>ti</td><td></td></tr>
<tr><td>eERK-KTR1.2/mScarlet-I/BCR.2a.ER-mTagBFP2</td><td>MKGRKPRDLELPLSPSLLGGQGPERTPGSGTSSGLQAPGPALSPSKRSGLEDDEPATPSKKPRTPSVSSRLERLTLQSSFQFPSGAPVSKGEAVIKEFMRFKVHMEGSMNGHEFEIEGEGEGRPYEGTQTAKLKVTKGGPLPFSWDILSPQFMYGSRAFIKHPADIPDYYKQSFPEGFKWERVMNFEDGGAVTVTQDTSLEDGTLIYKVKLRGTNFPPDGPVMQKKTMGWEASTERLYPEDGVLKGDIKMALRLKDGGRYLADFKTTYKAKKPVQMPGAYNVDRKLDITSHNEDYTVVEQYERSEGRHSTGGMDELYKGSGSAGGSAGGSDIEQELERAKASIRRLEQEVNQERSRMAYLQTLLAKGGSAGGSASGSGATNFSLLKQAGDVEENPG|<....>|PMKLSLVAAMLLLLSAARAVSKGEELIKENMHMKLYMEGTVDNHHFKCTSEGEGKPYEGTQTMRIKVVEGGPLPFAFDILATSFLYGSKTFINHTQGIPDFFKQSFPEGFTWERVTTYEDGGVLTATQDTSLQDGCLIYNVKIRGVNFTSNGPVMQKKTLGWEAFTETLYPADGGLEGRNDMALKLVGGSHLIANAKTTYRSKKPAKNLKMPGVYYVDYRLERIKEANNETYVEQHEVAVARYCDLPSKLGHKLNKDEL</td><td>pLenti</td><td>YG21</td></tr>
<tr><td>ePKA-KTR1.2/tdCherry.2a.3xNLS-mTagBFP2</td><td>MEMPEEPANSGHSLPPVYIYSPEYVSICDSLVKVPKRASMVNEDEAPSKRRASLGVSSRLERLTLQSSGAPVSKGEEDNMAIIKEFMRFKVHMEGSVNGHEFEIEGEGEGRPYEGTQTAKLKVTKGGPLPFAWDILSPQFMYGSKAYVKHPADIPDYLKLSFPEGFKWERVMNFEDGGVVTVTQDSSLQDGEFIYKVKLRGTNFPSDGPVMQKKTMGWEASSERMYPEDGALKGEIKQRLKLKDGGHYDAEVKTTYKAKKPVQLPGAYNVNIKLDITSHNEDYTIVEQYERAEGRHSTGGMDELYKARGSAGSATTMVSKGEEDNMAIIKEFMRFKVHMEGSVNGHEFEIEGEGEGRPYEGTQTAKLKVTKGGPLPFAWDILSPQFMYGSKAYVKHPADIPDYLKLSFPEGFKWERVMNFEDGGVVTVTQDSSLQDGEFIYKVKLRGTNFPSDGPVMQKKTMGWEASSERMYPEDGALKGEIKQRLKLKDGGHYDAEVKTTYKAKKPVQLPGAYNVNIKLDITSHNEDYTIVEQYERAEGRHSTGGMDELYKASGSGATNFSLLKQAGDVEENPG|<....>|PSPAPKKKRKVGDGGCPKKKRKVGDGGCPKKKRKVGDSSRVSKGEELIKENMHMKLYMEGTVDNHHFKCTSEGEGKPYEGTQTMRIKVVEGGPLPFAFDILATSFLYGSKTFINHTQGIPDFFKQSFPEGFTWERVTTYEDGGVLTATQDTSLQDGCLIYNVKIRGVNFTSNGPVMQKKTLGWEAFTETLYPADGGLEGRNDMALKLVGGSHLIANAKTTYRSKKPAKNLKMPGVYYVDYRLERIKEANNETYVEQHEVAVARYCDLPSKLGHKLNGMDELYKGRSPGLNGGSGATNFSLLKQAGDVEENPG</td><td>pC</td><td>S381</td></tr>
<tr><td>ePKA-KTR1.2/tdCherry.2a.ER-mTagBFP2</td><td>MEMPEEPANSGHSLPPVYIYSPEYVSICDSLVKVPKRASMVNEDEAPSKRRASLGVSSRLERLTLQSSGAPVSKGEEDNMAIIKEFMRFKVHMEGSVNGHEFEIEGEGEGRPYEGTQTAKLKVTKGGPLPFAWDILSPQFMYGSKAYVKHPADIPDYLKLSFPEGFKWERVMNFEDGGVVTVTQDSSLQDGEFIYKVKLRGTNFPSDGPVMQKKTMGWEASSERMYPEDGALKGEIKQRLKLKDGGHYDAEVKTTYKAKKPVQLPGAYNVNIKLDITSHNEDYTIVEQYERAEGRHSTGGMDELYKARGSAGSATTMVSKGEEDNMAIIKEFMRFKVHMEGSVNGHEFEIEGEGEGRPYEGTQTAKLKVTKGGPLPFAWDILSPQFMYGSKAYVKHPADIPDYLKLSFPEGFKWERVMNFEDGGVVTVTQDSSLQDGEFIYKVKLRGTNFPSDGPVMQKKTMGWEASSERMYPEDGALKGEIKQRLKLKDGGHYDAEVKTTYKAKKPVQLPGAYNVNIKLDITSHNEDYTIVEQYERAEGRHSTGGMDELYKASGSGATNFSLLKQAGDVEENPG|<....>|PMKLSLVAAMLLLLSAARAVSKGEELIKENMHMKLYMEGTVDNHHFKCTSEGEGKPYEGTQTMRIKVVEGGPLPFAFDILATSFLYGSKTFINHTQGIPDFFKQSFPEGFTWERVTTYEDGGVLTATQDTSLQDGCLIYNVKIRGVNFTSNGPVMQKKTLGWEAFTETLYPADGGLEGRNDMALKLVGGSHLIANAKTTYRSKKPAKNLKMPGVYYVDYRLERIKEANNETYVEQHEVAVARYCDLPSKLGHKLNKDEL</td><td>pLenti</td><td>YG63</td></tr>
<tr><td>ePKA-KTR1.2/tdTomato.2a.ER-mTagBFP2</td><td>MEMPEEPANSGHSLPPVYIYSPEYVSICDSLVKVPKRASMVNEDEAPSKRRASLGVSSRLERLTLQSSGAPVSKGEEVIKEFMRFKVRMEGSMNGHEFEIEGEGEGRPYEGTQTAKLKVTKGGPLPFAWDILSPQFMYGSKAYVKHPADIPDYKKLSFPEGFKWERVMNFEDGGLVTVTQDSSLQDGTLIYKVKMRGTNFPPDGPVMQKKTMGWEASTERLYPRDGVLKGEIHQALKLKDGGHYLVEFKTIYMAKKPVQLPGYYYVDTKLDITSHNEDYTIVEQYERSEGRHHLFLGHGTGSTGSGSSGTASSEDNNMAVIKEFMRFKVRMEGSMNGHEFEIEGEGEGRPYEGTQTAKLKVTKGGPLPFAWDILSPQFMYGSKAYVKHPADIPDYKKLSFPEGFKWERVMNFEDGGLVTVTQDSSLHDGTLIYKVKMRGTNFPPDGPVMQKKTMGWEASTERLYPRDGVLKGDIHQALKLKDGGHYLVEFKTIYMAKKPVQLPGYYYVDTKLDITSHNEDYTIVEQYERSEGRHHLFLYGMDELYKASGSGATNFSLLKQAGDVEENPG|<....>|PMKLSLVAAMLLLLSAARAVSKGEELIKENMHMKLYMEGTVDNHHFKCTSEGEGKPYEGTQTMRIKVVEGGPLPFAFDILATSFLYGSKTFINHTQGIPDFFKQSFPEGFTWERVTTYEDGGVLTATQDTSLQDGCLIYNVKIRGVNFTSNGPVMQKKTLGWEAFTETLYPADGGLEGRNDMALKLVGGSHLIANAKTTYRSKKPAKNLKMPGVYYVDYRLERIKEANNETYVEQHEVAVARYCDLPSKLGHKLNKDEL</td><td>pLenti</td><td>YG50</td></tr>
<tr><td>eERK-KTR1.2/emiRFP670.2a.ER-mTagBFP2</td><td>MKGRKPRDLELPLSPSLLGGQGPERTPGSGTSSGLQAPGPALSPSKRSGLEDDEPATPSKKPRTPSVSSRLERLTLQSSFQFPSGAPMAEGSVARQPDLLTCEHEEIHLAGSIQPHGALLVVSEHDHRVIQASANAAEFLNLGSVLGVPLAEIDGDLLIKILPHLDPTAEGMPVAVRCRIGNPSTEYCGLMHRPPEGGLIIELERAGPSIDLSGTLAPALERIRTAGSLRALCDDTVLLFQQCTGYDRVMVYRFDEQGHGLVFSECHVPGLESYFGNRYPSSTVPQMARQLYVRQRVRVLVDVTYQPVPLEPRLSPLTGRDLDMSGCFLRSMSPCHLQFLKDMGVRATLAVSLVVGGKLWGLVVCHHYLPRFIRFELRAICKRLAERIATRITALESLYKASGSGATNFSLLKQAGDVEENPG|<....>|PMKLSLVAAMLLLLSAARAVSKGEELIKENMHMKLYMEGTVDNHHFKCTSEGEGKPYEGTQTMRIKVVEGGPLPFAFDILATSFLYGSKTFINHTQGIPDFFKQSFPEGFTWERVTTYEDGGVLTATQDTSLQDGCLIYNVKIRGVNFTSNGPVMQKKTLGWEAFTETLYPADGGLEGRNDMALKLVGGSHLIANAKTTYRSKKPAKNLKMPGVYYVDYRLERIKEANNETYVEQHEVAVARYCDLPSKLGHKLNKDEL</td><td>pLenti</td><td>YG6</td></tr>
<tr><td>ePKA-KTR1.4/tdTomato.2a.ER-mTagBFP2</td><td>MEMPEEPANSGHSLPPVYIYSPEYVSICDSLVKVPKRASMVNEEDEAPAKRRASLGVSSRLERLTLQSSGAPVSKGEEVIKEFMRFKVRMEGSMNGHEFEIEGEGEGRPYEGTQTAKLKVTKGGPLPFAWDILSPQFMYGSKAYVKHPADIPDYKKLSFPEGFKWERVMNFEDGGLVTVTQDSSLQDGTLIYKVKMRGTNFPPDGPVMQKKTMGWEASTERLYPRDGVLKGEIHQALKLKDGGHYLVEFKTIYMAKKPVQLPGYYYVDTKLDITSHNEDYTIVEQYERSEGRHHLFLGHGTGSTGSGSSGTASSEDNNMAVIKEFMRFKVRMEGSMNGHEFEIEGEGEGRPYEGTQTAKLKVTKGGPLPFAWDILSPQFMYGSKAYVKHPADIPDYKKLSFPEGFKWERVMNFEDGGLVTVTQDSSLHDGTLIYKVKMRGTNFPPDGPVMQKKTMGWEASTERLYPRDGVLKGDIHQALKLKDGGHYLVEFKTIYMAKKPVQLPGYYYVDTKLDITSHNEDYTIVEQYERSEGRHHLFLYGMDELYKASGSGATNFSLLKQAGDVEENPG|<....>|PMKLSLVAAMLLLLSAARAVSKGEELIKENMHMKLYMEGTVDNHHFKCTSEGEGKPYEGTQTMRIKVVEGGPLPFAFDILATSFLYGSKTFINHTQGIPDFFKQSFPEGFTWERVTTYEDGGVLTATQDTSLQDGCLIYNVKIRGVNFTSNGPVMQKKTLGWEAFTETLYPADGGLEGRNDMALKLVGGSHLIANAKTTYRSKKPAKNLKMPGVYYVDYRLERIKEANNETYVEQHEVAVARYCDLPSKLGHKLNKDEL</td><td>pLenti</td><td>YG115</td></tr>
<tr><td>PKA-KTR2/mCherry.2a.H2B-</td><td>MVLRRASLGKRRASLGKRRASLGVDQLRLERLQIDEGAPVSKGEEDNMAIIKEFMRFKVHMEGSVNGHEFEIEGEGEGRPYEGTQTAKLKVTKGGPLPFAWDILSPQFMYGSKAYVKHPADIPDYLKLSFPEGFKWERVMNFEDGGVVTVTQDSSLQDGEFIYKVKLRGTNFPSDGPVMQKKTMGWEASSERMYPEDGALKGEIKQRLKLKDGGHYDAEVKTTYKAKKPVQLPGAYNVNIKLDITSHNEDYTIVEQYERAEGRHSTGGMDELYKASGSGATNFSLLKQAGDVEENPG|<....>|PSPAMPEPAKSAPAPKKGSKKAVTKAQKKGGKKRKRSRKESYSIYVYKVLKQVHPDTGISSKAMGIMNSFVNDIFERIAGEASRLAHYNK</td><td>pC</td><td>S737</td></tr>
</table>

bioRxiv preprint doi: https://doi.org/10.1101/2024.09.30.615856; this version posted October 2, 2024. The copyright holder for this preprint (which was not certified by peer review) is the author/funder, who has granted bioRxiv a license to display the preprint in perpetuity. It is made available under aCC-BY 4.0 International license.

**mTagBFP2**	RSTITSREIQTAVRLLLPGELAKHAVSEGTKAITKYTSAKDPGGGSSRVSKGEELIKENMHMKLYMEGTVDNHHFKCTSEGEGKPYEGTQTMRIKVVEGGPLPFAFDILATSFLYGSKTFINHTQGIPDFFKQSFPEGFTWERVTTYEDGGVLTATQDTSLQDGCLIYNVKIRGVNFTSNGPVMQKKTLGWEAFTETLYPADGGLEGRNDMALKLVGGSHLIANAKTTYRSKKPAKNLKMPGVYYVDYRLERIKEANNETYVEQHEVAVARYCDLPSKLGHKLNGMDELYKGRSPGLNGGSGATNFSLLKQAGDVEENPG		
**PKA-KTR2/tdCherry.2a.H2B-mTagBFP2**	**MVLRRASLGKRRASLGKRRASLGVDQLRLERLQIDE**GAPVSKGEEDNMAIIKEFMRFKVHMEGSVNGHEFEIEGEGEGRPYEGTQTAKLKVTKGGPLPFAWDILSPQFMYGSKAYVKHPADIPDYLKLSFPEGFKWERVMNFEDGGVVTVTQDSSLQDGEFIYKVKLRGTNFPSDGPVMQKKTMGWEASSERMYPEDGALKGEIKQRLKLKDGGHYDAEVKTTYKAKKPVQLPGAYNVNIKLDITSHNEDYTIVEQYERAEGRHSTGGMDELYKARGSAGSATTMVSKGEEDNMAIIKEFMRFKVHMEGSVNGHEFEIEGEGEGRPYEGTQTAKLKVTKGGPLPFAWDILSPQFMYGSKAYVKHPADIPDYLKLSFPEGFKWERVMNFEDGGVVTVTQDSSLQDGEFIYKVKLRGTNFPSDGPVMQKKTMGWEASSERMYPEDGALKGEIKQRLKLKDGGHYDAEVKTTYKAKKPVQLPGAYNVNIKLDITSHNEDYTIVEQYERAEGRHSTGGMDELYKASGSGATNFSLLKQAGDVEENPG**|<....>|**PSPAMPEPAKSAPAPKKGSKKAVTKAQKKGGKKRKRSRKESYSIYVYKVLKQVHPDTGISSKAMGIMNSFVNDIFERIAGEASRLAHYNKRSTITSREIQTAVRLLLPGELAKHAVSEGTKAITKYTSAKDPGGGSSRVSKGEELIKENMHMKLYMEGTVDNHHFKCTSEGEGKPYEGTQTMRIKVVEGGPLPFAFDILATSFLYGSKTFINHTQGIPDFFKQSFPEGFTWERVTTYEDGGVLTATQDTSLQDGCLIYNVKIRGVNFTSNGPVMQKKTLGWEAFTETLYPADGGLEGRNDMALKLVGGSHLIANAKTTYRSKKPAKNLKMPGVYYVDYRLERIKEANNETYVEQHEVAVARYCDLPSKLGHKLNGMDELYKGRSPGLNGGSGATNFSLLKQAGDVEENPG	**pC**	**S738**
**PKA-KTR3/mCherry.2a.ER-mTagBFP2**	**MEMPEEPANSGHSLPPVYIYSPEYSIFDSLVKVKRNPTVVNEDEAPSKRKRSLPISSRLERLTLQSS**GAPVSKGEEDNMAIIKEFMRFKVHMEGSVNGHEFEIEGEGEGRPYEGTQTAKLKVTKGGPLPFAWDILSPQFMYGSKAYVKHPADIPDYLKLSFPEGFKWERVMNFEDGGVVTVTQDSSLQDGEFIYKVKLRGTNFPSDGPVMQKKTMGWEASSERMYPEDGALKGEIKQRLKLKDGGHYDAEVKTTYKAKKPVQLPGAYNVNIKLDITSHNEDYTIVEQYERAEGRHSTGGMDELYKASGSGATNFSLLKQAGDVEENPG**|<....>|**PMKLSLVAAMLLLLSAARAVSKGEELIKENMHMKLYMEGTVDNHHFKCTSEGEGKPYEGTQTMRIKVVEGGPLPFAFDILATSFLYGSKTFINHTQGIPDFFKQSFPEGFTWERVTTYEDGGVLTATQDTSLQDGCLIYNVKIRGVNFTSNGPVMQKKTLGWEAFTETLYPADGGLEGRNDMALKLVGGSHLIANAKTTYRSKKPAKNLKMPGVYYVDYRLERIKEANNETYVEQHEVAVARYCDLPSKLGHKLNKDEL	**pLenti**	**YG69**
**PKA-KTR3/tdCherry.2a.ER-mTagBFP2**	**MEMPEEPANSGHSLPPVYIYSPEYSIFDSLVKVKRNPTVVNEDEAPSKRKRSLPISSRLERLTLQSS**GAPVSKGEEDNMAIIKEFMRFKVHMEGSVNGHEFEIEGEGEGRPYEGTQTAKLKVTKGGPLPFAWDILSPQFMYGSKAYVKHPADIPDYLKLSFPEGFKWERVMNFEDGGVVTVTQDSSLQDGEFIYKVKLRGTNFPSDGPVMQKKTMGWEASSERMYPEDGALKGEIKQRLKLKDGGHYDAEVKTTYKAKKPVQLPGAYNVNIKLDITSHNEDYTIVEQYERAEGRHSTGGMDELYKARGSAGSATTMVSKGEEDNMAIIKEFMRFKVHMEGSVNGHEFEIEGEGEGRPYEGTQTAKLKVTKGGPLPFAWDILSPQFMYGSKAYVKHPADIPDYLKLSFPEGFKWERVMNFEDGGVVTVTQDSSLQDGEFIYKVKLRGTNFPSDGPVMQKKTMGWEASSERMYPEDGALKGEIKQRLKLKDGGHYDAEVKTTYKAKKPVQLPGAYNVNIKLDITSHNEDYTIVEQYERAEGRHSTGGMDELYKASGSGATNFSLLKQAGDVEENPG**|<....>|**PMKLSLVAAMLLLLSAARAVSKGEELIKENMHMKLYMEGTVDNHHFKCTSEGEGKPYEGTQTMRIKVVEGGPLPFAFDILATSFLYGSKTFINHTQGIPDFFKQSFPEGFTWERVTTYEDGGVLTATQDTSLQDGCLIYNVKIRGVNFTSNGPVMQKKTLGWEAFTETLYPADGGLEGRNDMALKLVGGSHLIANAKTTYRSKKPAKNLKMPGVYYVDYRLERIKEANNETYVEQHEVAVARYCDLPSKLGHKLNKDEL	**pLenti**	**YG71**
**ERK-KTR1/emiRFP670.2a.ER-mTagBFP2**	**MKGRKPRDLELPLSPSLLGGQGPERTPGSGTSSGLQAPGPALSPSKRSGLEDPATPSKKPRTPSVSSRLERLTLQSSFQFPS**GAPMAEGSVARQPDLLTCEHEEIHLAGSIQPHGALLVVSEHDHRVIQASANAAEFLNLGSVLGVPLAEIDGDLLIKILPHLDPTAEGMPVAVRCRIGNPSTEYCGLMHRPPEGGLIIELERAGPSIDLSGTLAPALERIRTAGSLRALCDDTVLLFQQCTGYDRVMVYRFDEQGHGLVFSECHVPGLESYFGNRYPSSTVPQMARQLYVRQRVRVLVDVTYQPVPLEPRLSPLTGRDLDMSGCFLRSMSPCHLQFLKDMGVRATLAVSLVVGGKLWGLVVCHHYLPRFIRFELRAICKRLAERIATRITALESLYKASGSGATNFSLLKQAGDVEENPG**|<....>|**PMKLSLVAAMLLLLSAARAVSKGEELIKENMHMKLYMEGTVDNHHFKCTSEGEGKPYEGTQTMRIKVVEGGPLPFAFDILATSFLYGSKTFINHTQGIPDFFKQSFPEGFTWERVTTYEDGGVLTATQDTSLQDGCLIYNVKIRGVNFTSNGPVMQKKTLGWEAFTETLYPADGGLEGRNDMALKLVGGSHLIANAKTTYRSKKPAKNLKMPGVYYVDYRLERIKEANNETYVEQHEVAVARYCDLPSKLGHKLNKDEL	**pLenti**	**YG45**

bioRxiv preprint doi: https://doi.org/10.1101/2024.09.30.615856; this version posted October 2, 2024. The copyright holder for this preprint (which was not certified by peer review) is the author/funder, who has granted bioRxiv a license to display the preprint in perpetuity. It is made available under aCC-BY 4.0 International license.

Table S2. List of plasmid numbers and the ORF they express.

Name	Description (ORF expressed)	vector backbone	promoter
S739	PKA-KTR1-mCherry 2a H2B-mTagBFP2	pC (plasmid)	CMV
S777	mCherry-PKA-KTR2 2a H2B-mTagBFP2	pC (plasmid)	CMV
S740	PKA-KTR1-tdCherry 2a H2B-mTagBFP2	pC (plasmid)	CMV
S738	PKA-KTR2-tdCherry 2a H2B-mTagBFP2	pC (plasmid)	CMV
S741	ePKA-KTR1.1-tdCherry 2a H2B-mTagBFP2	pC (plasmid)	CMV
S467	ePKA-KTR1.2-tdCherry 2a H2B-mTagBFP2	pC (plasmid)	CMV
S514	ePKA-KTR1.3-tdCherry 2a H2B-mTagBFP2	pC (plasmid)	CMV
S515	ePKA-KTR1.4-tdCherry 2a H2B-mTagBFP2	pC (plasmid)	CMV
S742	ePKA-KTR1.5-tdCherry 2a H2B-mTagBFP2	pC (plasmid)	CMV
YG63	ePKA-KTR1.2-tdCherry 2a SS-mTagBFP2-KDEL	pLenti (lentiviral vector)	CMV
S381	ePKA-KTR1.2-tdCherry 2a 3xNLS-mTagBFP2	pC (plasmid)	CMV
YG61	ePKA-KTR1.2-mCherry 2a SS-mTagBFP2-KDEL	pLenti (lentiviral vector)	CMV
YG65	ePKA-KTR1.4-mCherry 2a SS-mTagBFP2-KDEL	Lentiviral	CMV
YG67	ePKA-KTR1.4-tdCherry 2a SS-mTagBFP2-KDEL	Lentiviral	CMV
YG8	ePKA-KTR1.2-mScarlet 2a SS-mTagBFP2-KDEL	Lentiviral	CMV
YG18	ePKA-KTR1.2-mScarlet-BCR 2a SS-mTagBFP2-KDEL	Lentiviral	CMV
S468	ERK-KTR1-tdCherry 2a H2B-mTagBFP2	pC (plasmid)	CMV
S712	eERK-KTR1.1-tdCherry 2a H2B-mTagBFP2	pC (plasmid)	CMV
S713	eERK-KTR1.2-tdCherry 2a H2B-mTagBFP2	pC (plasmid)	CMV
S765	eERK-KTR1.3-tdCherry 2a H2B-mTagBFP2	pC (plasmid)	CMV
S940	eERK-KTR1.2-mScarlet 2a SS-mTagBFP2-KDEL	Lentiviral	CMV
YG21	eERK-KTR1.2-mScarlet-BCR 2a SS-mTagBFP2-KDEL	Lentiviral	CMV
YG6	eERK-KTR1.2-emiRFP670 2a SS-mTagBFP2-KDEL	pLenti (lentiviral vector)	CMV
YG45	ERK-KTR1-emiRFP670 2a SS-mTagBFP2-KDEL	pLenti (lentiviral vector)	CMV
YG50	ePKA-KTR1.2-tdTomato 2a SS-mTagBFP2-KDEL	pLenti (lentiviral vector)	CMV
YG114	PKA-KTR3-tdTomato 2a SS-mTagBFP2-KDEL	pLenti (lentiviral vector)	CMV
YG115	ePKA-KTR1.4-tdTomato 2a SS-mTagBFP2-KDEL	pLenti (lentiviral vector)	CMV
S1137	jGCaMP8s	Lentiviral	CMV
YG160	eERK-KTR1.2(mutated)-emiRFP670 2a SS-mTagBFP2-KDEL	pLenti (lentiviral vector)	CMV

bioRxiv preprint doi: https://doi.org/10.1101/2024.09.30.615856; this version posted October 2, 2024. The copyright holder for this preprint (which was not certified by peer review) is the author/funder, who has granted bioRxiv a license to display the preprint in perpetuity. It is made available under aCC-BY 4.0 International license.

Structured Methods - Reagents and Tools Table

Instructions: Please complete the relevant fields below, adding rows as needed. The following page provides an example of a completed table and additional instruction for entering your data in the table.

Reagent/Resource	Reference or Source	Identifier or Catalog Number
**Experimental Models**		
HEK-293 cells (H. sapiens)	ATCC	CRL-1573
Dorsal root ganglion neurons	C57BL/6 (Jackson Lab)	000664
**Recombinant DNA**		
psPAX2	Addgene	12260
VSV.G	Addgene	14888
PKA-KTR1/tdCherry.2a.H2B-mTagBFP2	This study	N/A
PKA-KTR1/mCherry.2a.H2B-mTagBFP2	This study	N/A
ePKA-KTR1.1/tdCherry.2a.H2B-mTagBFP2	This study	N/A
ePKA-KTR1.2/tdCherry.2a.H2B-mTagBFP2	This study	N/A
ePKA-KTR1.3/tdCherry.2a.H2B-mTagBFP2	This study	N/A
ePKA-KTR1.4/tdCherry.2a.H2B-mTagBFP2	This study	N/A
ePKA-KTR1.5/tdCherry.2a.H2B-mTagBFP2	This study	N/A
ePKA-KTR1.2/mCherry.2a.ER-mTagBFP2	This study	N/A
ePKA-KTR1.4/mCherry.2a.ER-mTagBFP2	This study	N/A
ePKA-KTR1.4/tdCherry.2a.ER-mTagBFP2	This study	N/A

bioRxiv preprint doi: https://doi.org/10.1101/2024.09.30.615856; this version posted October 2, 2024. The copyright holder for this preprint (which was not certified by peer review) is the author/funder, who has granted bioRxiv a license to display the preprint in perpetuity. It is made available under aCC-BY 4.0 International license.

ePKA-KTR1.2/mScarlet-I.2a.ER-mTagBFP2	This study	N/A
ePKA-KTR1.2/mScarlet-I/BCR.2a.ER-mTagBFP2	This study	N/A
ERK-KTR1/tdCherry.2a.H2B-mTagBFP2	This study	N/A
eERK-KTR1.1/tdCherry.2a.H2B-mTagBFP2	This study	N/A
eERK-KTR1.2/tdCherry.2a.H2B-mTagBFP2	This study	N/A
eERK-KTR1.3/tdCherry.2a.H2B-mTagBFP2	This study	N/A
eERK-KTR1.2/mScarlet-I.2a.ER-mTagBFP2	This study	N/A
eERK-KTR1.2/mScarlet-I/BCR.2a.ER-mTagBFP2	This study	N/A
ePKA-KTR1.2/tdCherry.2a.3xNLS-mTagBFP2	This study	N/A
ePKA-KTR1.2/tdCherry.2a.ER-mTagBFP2	This study	N/A
ePKA-KTR1.2/tdTomato.2a.ER-mTagBFP2	This study	N/A
eERK-KTR1.2/emiRFP670.2a.ER-mTagBFP2	This study	N/A
ePKA-KTR1.4/tdTomato.2a.ER-mTagBFP2	This study	N/A
PKA-KTR2/mCherry.2a.H2B-mTagBFP2	This study	N/A
PKA-KTR2/tdCherry.2a.H2B-mTagBFP2	This study	N/A
PKA-KTR3/mCherry.2a.ER-mTagBFP2	This study	N/A
PKA-KTR3/tdCherry.2a.ER-mTagBFP2	This study	N/A

bioRxiv preprint doi: https://doi.org/10.1101/2024.09.30.615856; this version posted October 2, 2024. The copyright holder for this preprint (which was not certified by peer review) is the author/funder, who has granted bioRxiv a license to display the preprint in perpetuity. It is made available under aCC-BY 4.0 International license.

ERK-KTR1/emiRFP670.2a.ER-mTagBFP2	This study	N/A
Additional plasmids and more information	This study	Table S1
**Antibodies**		
Rabbit anti-phospho-ERK1/2	Cell Signaling Technology	9101S
Rabbit anti-phospho-PKA R2 (Ser99)	Abcam	ab32390
Goat anti-rabbit Alexa488	Jackson ImmunoResearch	AB_2338046
**Oligonucleotides and other sequence-based reagents**		
Geneblocks and primers	Integrated DNA Technologies	Table S1
**Chemicals, Enzymes and other reagents**		
Phusion High-Fidelity DNA Polymerase	ThermoFisher	F530L
T4 DNA Ligase (5 U/µL)	ThermoFisher	EL0011
Petri Dishes	Falcon	351029
DMEM, high glucose	ThermoFisher	11965118
Trypsin-EDTA (0.25%), phenol red	ThermoFisher	25200114
Fetal Bovine Serum	ThermoFisher	16000044
24-well cell culture plates	Denville	1156F00
TC-treated Culture Dish (100 mm)	Corning	430167
Hanks' Balanced Salt Solution (HBSS)	ThermoFisher	14175103
96 Well Black Plate, Coverglass Bottom, CC2 Surface	ThermoFisher	160376
µ-Slide 8 Well high Glass Bottom	Ibidi	80807
µ-Slide 18 Well Glass Bottom	Ibidi	81817
A23187	Sigma-Aldrich	C7522
ATP	Sigma-Aldrich	A26209
Forskolin	Cayman Chemical	11018
H89	MedChemExpress	HY-15979A
EGF	Gibco	PHG0311L

bioRxiv preprint doi: https://doi.org/10.1101/2024.09.30.615856; this version posted October 2, 2024. The copyright holder for this preprint (which was not certified by peer review) is the author/funder, who has granted bioRxiv a license to display the preprint in perpetuity. It is made available under aCC-BY 4.0 International license.

VX-11e	SelleckChem	S7709
leptomycin B	Sigma-Aldrich	L2913
Phorbol 12-myristate 13-acetate	Sigma-Aldrich	P1585
Neurobasal™ Plus Medium	Gibco	A3582901
B-27™ Plus Supplement	Gibco	A3582801
Poly-L-lysine solution	Sigma-Aldrich	P8920-100ML
Laminin	Sigma-Aldrich	L2020
Papain, suspension	Worthington	LS003126
Bovine Serum Albumin	Sigma-Aldrich	A-9418
TM Liberase	Roche	5401119001
TL Liberase	Roche	5401020001
Pen/Strep (100X)	ThermoFisher	15140122
Lipofectamine™ 3000	ThermoFisher	L3000015
Open-Top Thinwall Ultra-Clear Tube	Beckman-Coulter	C14292
50 mL Luer-Lok™ Syringe	BD	309653
Syringe Filter (0.45 μm)	Millipore	SLHVR33RS
8-mm cover glasses	Electron Microscopy Sciences	7229608
QIAprep Spin Miniprep Kit	Qiagen	27106
Wizard® Plus Midipreps DNA Purification System	Promega	A7640
Ampicillin sodium salt	Sigma-Aldrich	A8351
**Software**		
GraphPad Prism 10	https://www.graphpad.com/	
SnapGene 7	https://www.snapgene.com/	
ImageJ (Fiji)	https://fiji.sc/	
NIS Elements Advanced Research v4.60	https://www.microscope.healthcare.nikon.com/products/software/nis-elements	
EVOS M7000 Cell Imaging System Software	https://www.thermofisher.com/us/en/home/technical-resources/software-downloads/evos-m7000-imaging-system-software-download.html	
**Other**		
EVOS M7000 Imaging System	ThermoFisher	AMF7000

bioRxiv preprint doi: https://doi.org/10.1101/2024.09.30.615856; this version posted October 2, 2024. The copyright holder for this preprint (which was not certified by peer review) is the author/funder, who has granted bioRxiv a license to display the preprint in perpetuity. It is made available under aCC-BY 4.0 International license.

EVOS Onstage Incubator	ThermoFisher	AMC2000
Optima L-90K ultracentrifuge	Beckman-Coulter	
SW-32 Ti rotor	Beckman-Coulter	
Sony MA900 Cell Sorter	Sony	
